# Differential Control of Asexual Development and Sterigmatocystin Biosynthesis by a Novel Regulator in *Aspergillus nidulans*

**DOI:** 10.1038/srep46340

**Published:** 2017-04-19

**Authors:** Yong Jin Kim, Yu Yeong Man, Pil Jae Maeng

**Affiliations:** 1 Department of Microbiology and Molecular Biology, Chungnam National University, Daejeon 34134, Korea

## Abstract

The filamentous fungus *Aspergillus nidulans* primarily reproduces by forming asexual spores called conidia and produces the mycotoxin sterigmatocystin (ST), the penultimate precursor of aflatoxins. It has been known that asexual development and ST production are tightly co-regulated by various regulatory inputs. Here, we report that the novel regulator AslA with a C_2_H_2_ domain oppositely regulates development and ST biosynthesis. Nullifying *aslA* resulted in defective conidiation and reduced expression of *brlA* encoding a key activator of asexual development, which indicates that AslA functions as an upstream activator of *brlA* expression. *aslA* deletion additionally caused enhanced ST production and expression of *aflR* encoding a transcriptional activator for ST biosynthetic genes, suggesting that AslA functions as an upstream negative regulator of *aflR*. Cellular and molecular studies showed that AslA has a trans-activation domain and is localized in the nuclei of vegetative and developing cells but not in spores, indicating that AslA is likely a transcription factor. Introduction of the *aslA* homologs from distantly-related aspergilli complemented the defects caused by *aslA* null mutation in *A. nidulans*, implying a functional conservancy of AslA. We propose that AslA is a novel regulator that may act at the split control point of the developmental and metabolic pathways.

The ascomycete fungus *Aspergillus nidulans* serves as one of the best model organisms for investing many aspects of cell biology and genetics of filamentous fungi, owing to the extensive available background information on its genetic and biochemical properties. *A. nidulans* has two major reproductive cycles, asexual and sexual, involving a number of developmental events, including spatiotemporal control of transcription for many genes, specialized cellular differentiation and intercellular communication.

During the asexual life cycle, conidial germination and vegetative growth yield undifferentiated networks of interconnected hyphal cells termed mycelium. After acquisition of developmental competency, asexual development is driven by a series of morphogenetic differentiation processes triggered by environmental signals, such as exposure to air^1^,^2^,^3^ and nutrient starvation^4^, to yield a specialized conidia*-*bearing structure known as the conidiophore. The central developmental pathway (CDP) controlling the temporal and spatial expression of conidiation specific genes involves three major regulatory transcription factors (TFs), BrlA, AbaA and WetA^5^. BrlA functions as a key transcriptional activator of the central regulatory pathway by directing the expression of other genes required for conidiation^5^,^6^,^7^. AbaA mainly controls the genes required for the middle and terminal stages of conidiophore development, including phialide formation^8^,^9^. WetA is responsible for activating the genes involved in conidial wall assembly^10^,^11^.

Expression of *brlA* is regulated by the upstream developmental activator (UDA) pathway consisting of FluG^12^,^13^, suppressors of *fluG* (SFGs, including SfgA^14^,^15^), and fluffy low *brlA* expression (FLBs, such as FlbA, FlbB, FlbC, FlbD and FlbE^3^). FluG functions in the synthesis of the extracellular signaling molecule directing asexual sporulation and potentially other aspects of colony growth^16^. The signal generated by FluG suppresses the expression of *sfgA* encoding the upstream negative regulator, resulting in derepression of *flb* gene expression. FlbA inhibits vegetative growth signaling mediated by a heterotrimeric G protein composed of FadA and SfaD::GpgA^17^,^18^, and is indirectly involved in the positive regulation of asexual development mediated by *brlA*^17^. FlbC is a putative C_2_H_2_ TF that directly controls the expression of *brlA*^19^. FlbB and FlbD, bZIP- and cMyb-type TFs, respectively, function cooperatively to activate *brlA* expression^20^,^21^. In addition, FlbE, a UDA containing two conserved uncharacterized domains, physically interacts with FlbB, and both proteins activate *flbD* expression interdependently^22^,^23^. In addition to the FluG-initiated UDA network, several negative regulators are involved in CDP gene expression. NsdD, a major zinc finger GATA-type activator of sexual reproduction, functions as a key negative regulator of conidiation, potentially exerting a repressive role via downregulating *brlA* expression^24^. The velvet protein, VeA, which functions as a pivotal positive regulator of sexual development, is also involved in negative control of asexual development^25^.

Fungi produce a variety of secondary metabolites, such as mycotoxins, antibiotics, pigments and sporulation-activating compounds. The molecular mechanisms controlling secondary metabolism are frequently involved in the regulation of asexual and sexual development^26^. One of the predominant regulatory links is the heterodimeric complex VelB-VeA, which associates with LaeA in the nucleus^27^,^28^. The resulting heterotrimeric VelB-VeA-LaeA complex coordinates secondary metabolism and development in the dark. The nuclear protein LaeA was the first-identified transcriptional activator of several secondary metabolite gene clusters in *A. nidulans* and is well conserved across fungi^29^,^30^. LaeA contains an S-adenosylmethionine (SAM) binding motif and appears to methylate histone proteins differentially to alter the chromatin structure for promoting gene expression^31^. The secondary metabolite gene clusters subjected to LaeA-dependent regulation contains the genes involved in biosynthesis of sterigmatocystin (ST), such as *aflR* and *stcA-X*^32^,^33^,^34^; and terraquinone (TQ), such as *tdiA-E*^30^,^35^.

In the present study, we characterized *aslA (as*exual differentiation with *l*ow-level conidiation, *AN5583*) gene encoding a C_2_H_2_-type zinc finger TF in relation to asexual development and secondary metabolism. We have previously analyzed the function of *aslA* with regard to K^+^ stress resistance and vacuolar morphology, and found that AslA attenuates the K^+^ stress-inducible expression of the genes involved in vacuolar sequestration of K^+^ ions and vacuolar biogenesis^36^. In this paper, we describe the characterization of the putative C_2_H_2_-type zinc finger transcription factor, AslA, in relation to both asexual differentiation and secondary metabolism. We provide evidence that AslA is required to activate asexual differentiation via positive regulation of the key conidiation-specific CDP gene, *brlA*. With regard to secondary metabolism, we found that AslA negatively controls the expression of the genes involved in secondary metabolite biosynthesis, such as ST and TQ. Introduction of the *A. fumigatus* or *A. flavus* orthologs of *aslA* into the Δ*aslA* strain led to recovery of the wild-type (WT) phenotypes related to asexual development and secondary metabolite production, implying functional conservation of *aslA* among the aspergilli.

## Results

### *aslA* is required for proper asexual development

**Figure 1:**
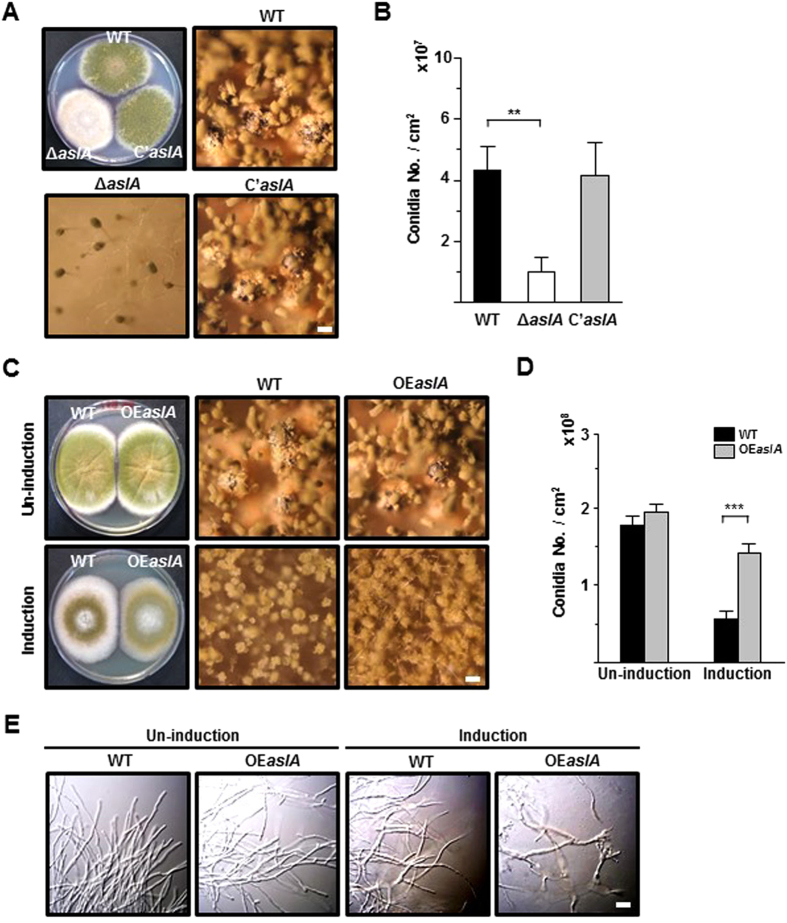
*aslA* is required for proper asexual development. (**A**) Colonies of the WT (MCBA003), Δ*aslA* (MCBA103) and *aslA* complementation (*C*’*aslA*, MCBA203) strains grown for 4 days after point-inoculation on solid MMG. Entire colonies and close-up views of the center of individual colonies are shown. Bar, 100 μm. (**B**) Quantitative analyses of conidia formation by the strains shown in (**A**) performed in triplicate (***P < 0.001). (**C**) Colonies of the WT and *OEaslA* (MCBA303) strains grown for 4 days after point-inoculation on solid MMG (Un-induction) and MMT (Induction). Entire colonies and close-up views of the center of individual colonies are shown. Bar, 100 μm. The result of Southern blot verifying a single copy integration of *aslA-*overexpression cassette into the *pyroA* locus of the *OEaslA* strain is presented in Supplementary Figure S1A-C online. The result of RT-qPCR supporting overexpression of *aslA* in the *OEaslA* strain is presented in Supplementary Figure S1D. (**D**) Quantitative analyses of conidia formation by the strains shown in (**C**) performed in triplicate (***P < 0.001). (**E**) Photomicrographs of the WT and *OEaslA* hyphae at 12 h post-transfer to MMG (Un-induction) and MMT (Induction). Bar, 20 μm.

To clarify the role of *aslA* gene in asexual development, we monitored the growth and development of the Δ*aslA* mutant (MCBA103) on glucose minimal medium (MMG) plates, compared with those of WT (MCBA003) and *aslA* complementation (*C*’*aslA*, MCBA203) strains. After 5-day culture on MMG, the Δ*aslA* mutant showed a similar rate of radial growth as the WT and *C*’*aslA* strains, but formed a fluffy-looking colony distinct from the two other strains that generated well-conidiated colonies (Fig. 1A). Accordingly, the number of conidia produced by the Δ*aslA* mutant was less than a quarter of those generated by the WT and *C*’*aslA* strains (Fig. 1B).

The effect of *aslA* overexpression on growth and development was determined by analyzing the phenotype of the *OEaslA* (MCBA303) strain. Following point inoculation and culture growth for 5 days on threonine minimal medium (MMT) plates, whereby *aslA* expression was induced by threonine, the *OEaslA* strain formed a fully conidiated colony while the WT strain formed an insufficiently developed colony (Fig. 1C). The number of conidia formed in the induced colony of the overexpression strain was more than two-fold higher than that of the WT strain (Fig. 1D). On the other hand, no significant phenotypic differences were observed between the *aslA* overexpression and WT strains on MMG. The effect of *aslA* overexpression was additionally assessed in liquid submerged culture, which normally suppresses sporulation. Interestingly, when mycelia of the overexpression strain grown for 18 h in liquid MMG were shifted for 12 h to liquid MMT, almost fully developed conidiophores were generated, in contrast to the WT strain (Fig. 1E). Taken together, these results clearly suggest that *aslA* contributes to the process of asexual development.

### AslA positively controls expression of the key CDP gene, *brlA*

**Figure 2:**
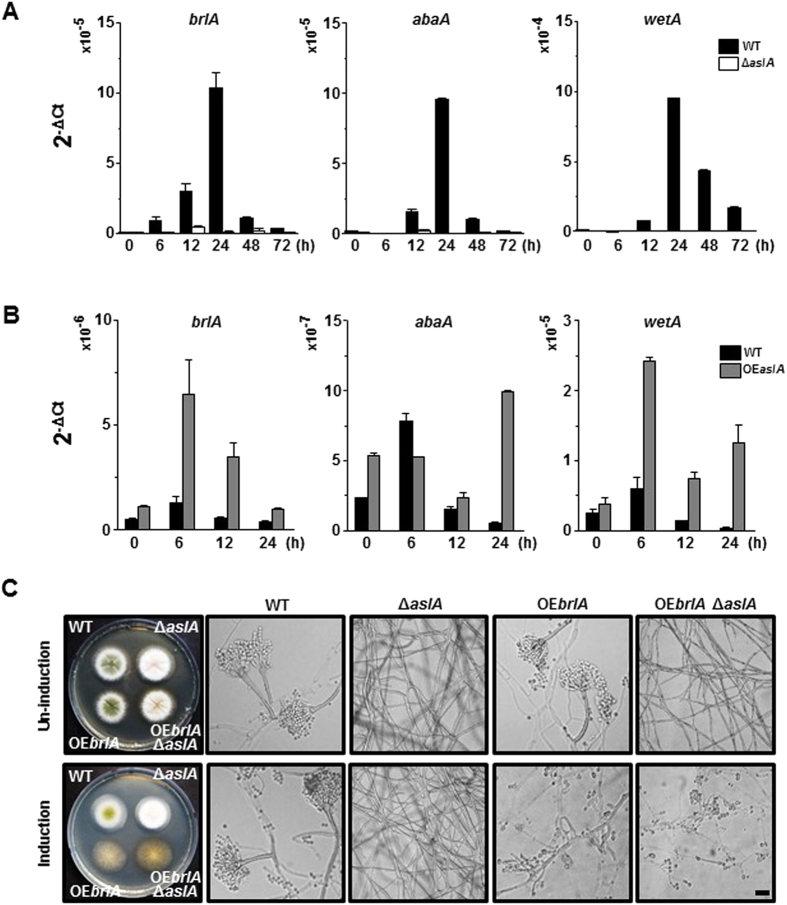
AslA positively control the expression of *brlA*. (**A** and **B**) RT-qPCR analyses of *brlA, abaA* and *wetA* mRNA levels in the WT (MCBA003), Δ*aslA* (MCBA103) and *OEaslA* (MCBA305) strains performed in triplicate. Mycelia of the strains grown in liquid MMG for 18 h were shifted to solid MMG (**A**) or MMT (**B**), and total RNAs were extracted after the time intervals indicated. Primers used for RT-qPCR: *brlA*, PbrlA-qf and PbrlA-qr; *abaA*, PabaA-qf and PabaA-qr; *wetA*, PwetA-qf and PwetA-qr; 18S rRNA (internal control), P18S-rRNA-qf and P18S-rRNA-qr. (**C**) Colony and hyphal morphology of the WT, Δ*aslA, OEaslA* and *OEbrlA* Δ*aslA* (MCBA553) strains. Colonies were grown for 3 days after point-inoculation on solid MMG (Un-induction) and MMT (Induction). For DIC microscopic observation of hyphae, each strain was coverslip-cultured on a block of MMG and MMT for 4 days. Bar, 20 μm. The results of Southern blot verifying single copy integrations of *brlA-*overexpression cassettes into the *pyroA* loci of the *OEbrlA* and *OEbrlA* Δ*aslA* strains are presented in Supplementary Figure S2A–C. The results of RT-qPCR supporting overexpression of *brlA* in the *OEbrlA* and *OEbrlA* Δ*aslA* strains are presented in Supplementary Figure S2D.

The above phenotypic traits of the Δ*aslA* mutant support the view that AslA transcriptionally regulates CDP genes controlling the conidiation-specific gene expression and the order of gene activation during conidiophore development and spore maturation. We thus monitored the changes of *brlA, abaA* and *wetA* mRNA levels during asexual development in the Δ*aslA* and *OEaslA* strains, compared with the WT strain, via real-time reverse transcription polymerase chain reaction (RT-qPCR). When mycelia grown in liquid MMG were shifted to solid MMG to induce synchronized asexual differentiation, the Δ*aslA* strain showed significantly reduced transcript levels of *brlA, abaA* and *wetA* compared to the WT strain (Fig. 2A). Upon inducing *aslA* expression by transferring mycelia of the *OEaslA* strain grown in liquid MMG to MMT, mRNA levels of the CDP genes were significantly increased (Fig. 2B). Considering that BrlA consecutively activates the downstream CDP genes, *abaA* and *wetA*^5^, the collective results suggest that AslA functions as a positive regulator of *brlA* expression, either directly or indirectly via interaction with other specific regulators.

To further elucidate the functional relationship between *aslA* and *brlA*, we observed the effect of *brlA* overexpression on the asexual differentiation-related phenotype induced by *aslA* deletion using the *OEbrlA* strain with Δ*aslA* or *aslA*^+^ background. On solid MMT, the *OEbrlA* Δ*aslA* (MCBA553) strain showed similar morphological features to the *OEbrlA* (MCBA353) strain, generating a characteristic colony bearing conidia at the tips of both branched aerial and substrate mycelium (Fig. 2C). Our results are consistent with previous reports that overexpression of *brlA* leads to termination of vegetative growth and formation of conidial spores from hyphae in submerged culture^3^. It appears that the fluffy phenotype resulting from *aslA* deletion is suppressed by *brlA* overexpression, in keeping with the view that *brlA* is located downstream of and positively controlled by *aslA*.

### The fluffy phenotype of Δ*aslA* is suppressed by Δ*nsdD* or *veA1* mutation

**Figure 3:**
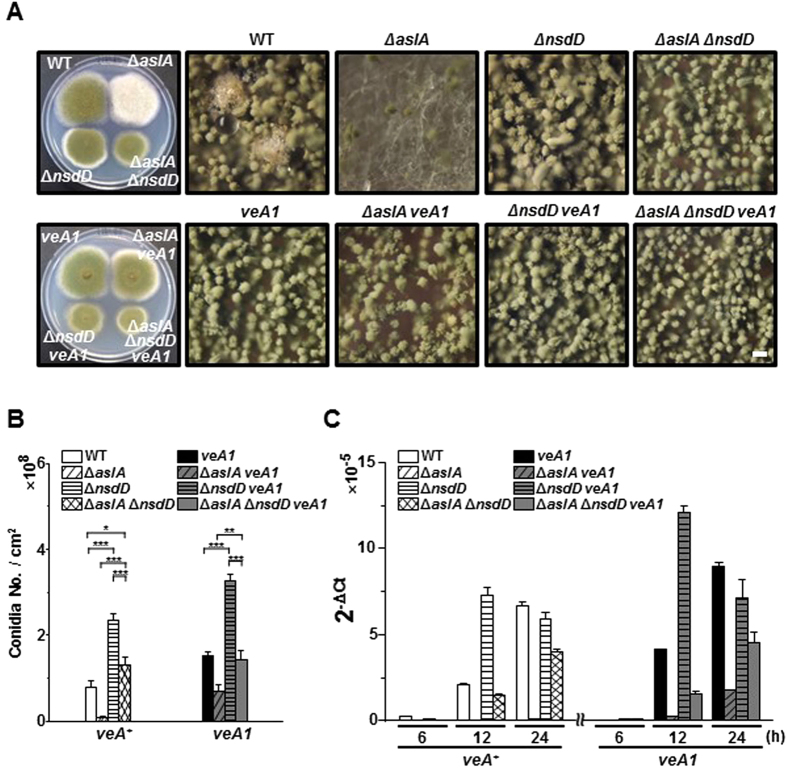
The fluffy phenotype of Δ*aslA* is suppressed by either Δ*nsdD* or *veA1* mutation. (**A**) Colony morphology of the WT (MCBA003), Δ*aslA* (MCBA103), Δ*nsdD* (TNJ108), Δ*aslA* Δ*nsdD* (MCBA403), *veA1* (MCBA004), Δ*aslA veA1* (MCBA104), Δ*nsdD veA1* (TNJ111) and Δ*aslA* Δ*nsdD veA1* (MCBA404) strains. Colonies were grown for 4 days after point-inoculation on solid MMG. Entire colonies and close-up views of the center of individual colonies are shown. Bar, 100 μm. (**B**) Quantitative analyses of conidiation by the strains shown in (**A**) performed in triplicate (***P < 0.001). (**C**) RT-qPCR analyses of *brlA* mRNA levels in the strains shown in (A) performed in triplicate. Mycelia of the strains grown in liquid MMG for 18 h were shifted to solid MMG, and total RNAs were extracted after the time intervals indicated. Primers used for RT-qPCR: *brlA*, PbrlA-qf and PbrlA-qr; 18S rRNA (internal control), P18S-rRNA-qf and P18S-rRNA-qr.

NsdD, a pivotal activator of sexual reproduction, also acts as a key negative regulator of conidiation, possibly exerting its repressive role through downregulation of *brlA*^24^. To assess the relationship between *aslA* and *nsdD*, we compared the phenotypes of the Δ*aslA* Δ*nsdD* double mutant (MCBA403) related to asexual differentiation, with those of the Δ*aslA*, Δ*nsdD* (TNJ108) and WT strains. When grown on solid MMG, the Δ*aslA* Δ*nsdD* and Δ*nsdD* strains formed well-conidiated colonies (Fig. 3A). Quantitative estimation of conidia formation disclosed that the Δ*nsdD* mutant forms more than twice as many conidia as WT while the Δ*aslA* Δ*nsdD* mutant produces similar amounts of conidia as the WT strain (Fig. 3B). We also analyzed the expression of *brlA* in the Δ*aslA* Δ*nsdD* mutant during asexual development in comparison with those of the Δ*aslA*, Δ*nsdD* and WT strains via RT-qPCR. During the period of synchronized asexual differentiation, the Δ*nsdD* mutant exhibited obviously increased and accelerated *brlA* expression compared to the WT strain. The Δ*aslA* Δ*nsdD* double mutant showed significantly increased level of *brlA* mRNA compared to the Δ*aslA* strain, hence up to more than 50% of that observed in the WT strain (Fig. 3C). Thus, we suggest that *nsdD* deletion suppresses the impaired conidiation phenotype caused by *aslA* deletion through derepression of *brlA* expression.

VeA, a major light-dependent regulator governing development in *A. nidulans*, also negatively controls *brlA* expression^27^. The relationship between *aslA* and *veA* was investigated by characterizing the asexual differentiation-related phenotype of the Δ*aslA veA1* (MCBA104) strain relative to those of the Δ*aslA, veA1* (MCBA004) and WT strains. The Δ*aslA veA1* strain produced a relatively well-conidiated colony bearing a similar number of conidia as WT, but generated half of the conidia formed by the *veA1* strain (Fig. 3A and B). Additionally, the level of *brlA* expression in the Δ*aslA veA1* mutant were apparently higher compared to that in the Δ*aslA* strain, although significantly lower than that observed in the WT strain (Fig. 3C). We suggest that, similarly as in the case of Δ*nsdD, veA1* mutation suppresses the effect of Δ*aslA* via derepression of *brlA* expression.

### AslA negatively regulates the expression of the genes necessary for ST and TQ biosynthesis

**Figure 4:**
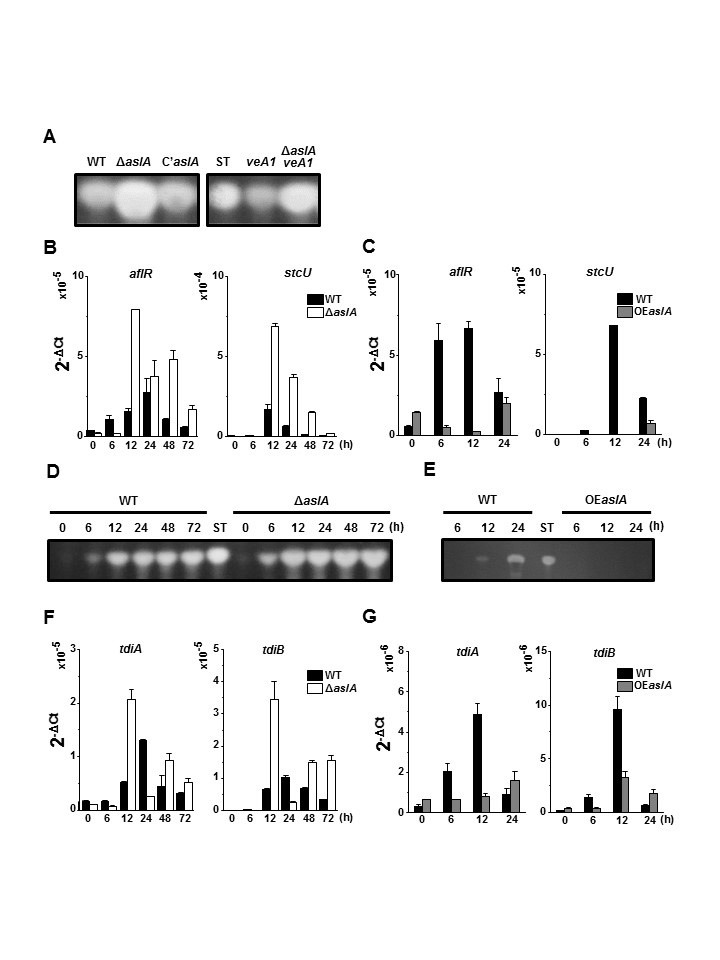
AslA negatively regulates the expression of the genes necessary for ST and TQ biosynthesis. (**A**) TLC analyses of ST in the chloroform extracts of the WT (MCBA003), Δ*aslA* (MCBA103), *C*’*aslA* (MCBA203), *veA1* (MCBA004) and Δ*aslA veA1* (MCBA104) strains. Colonies were grown for 5 days after point-inoculation on solid MMG, and 0.2 g (wet weight) of mycelium was used to prepare 100 μl of chloroform extract as described in Methods section. Approximately 20 μl of each sample and ST standard (5 mg) were loaded onto a TLC silica plate. ST, ST standard. Full-length TLC plates are presented in Supplementary Figure S3A. (**B**) and (**C**) RT-qPCR analyses of *aflR* and *stcU* mRNA levels in the WT (MCBA003), Δ*aslA* (MCBA103) and *OEaslA* (MCBA303) strains performed in triplicate. Mycelia of the strains grown in liquid MMG for 18 h were shifted to solid MMG (**B**) or MMT (**C**), and total RNAs were extracted after the time intervals indicated. Primers used for RT-qPCR: *aflR*, PaflR-qf and PaflR-qr; *stcU*, PstcU-qf and PstcU-qr; 18S rRNA (internal control), P18S-rRNA-qf and P18S-rRNA-qr. (**D** and **E**) TLC analyses of ST in the chloroform extracts of the WT, Δ*aslA* and *OEaslA* strains. Mycelia of the strains grown in liquid MMG for 18 h were shifted to solid MMG (**D**) or MMT (**E**), and 0.2 g (wet weight) of mycelium was used to prepare 100 μl of chloroform extract as described in Methods section after the time intervals indicated. Approximately 20 μl of each sample and ST standard (5 mg) were loaded onto a TLC silica plate. ST, ST standard. Full-length TLC plates are presented in Supplementary Figure S3B and C. (**F**) and (**G**) RT-qPCR analyses of *tdiA* and *tdiB* mRNA levels in the WT, Δ*aslA* and *OEaslA* strains performed in triplicate. Mycelia of the strains grown in liquid MMG for 18 h were shifted to solid MMG (**F**) or MMT (**G**), and total RNAs were extracted after the time intervals indicated. Primers used for RT-qPCR: *tdiA*, PtdiA-qf and PtdiA-qr; *tdiB*, PtdiB-qf and PtdiB-qr; 18S rRNA (internal control), P18S-rRNA-qf and P18S-rRNA-qr.

In general, the molecular mechanism of *A. nidulans* development is closely related to secondary metabolism. To ascertain whether *aslA* plays a role in secondary metabolism, we initially assessed the effect of *aslA* deletion on ST biosynthesis via thin-layer chromatography (TLC) analysis. In both *veA*^+^ and *veA1* background, the Δ*aslA* mutant produced increased amount of ST, compared with the WT and *C*’*aslA* strains inoculated and grown on solid MMG for 5 days (Fig. 4A). Additionally, we monitored the time-course profile of ST production and expression levels of genes involved in ST biosynthesis, such as *aflR* and *stcU*. When mycelia grown in liquid MMG were shifted to solid MMG, the Δ*aslA* mutant showed significantly increased *aflR* and *stcU* expression (Fig. 4B) as well as ST production (Fig. 4D) throughout asexual development compared to the WT strain. The effects of *aslA* overexpression on ST biosynthesis were assessed using the *OEaslA* strain. Significantly decreased levels of *aflR* and *stcU* expression were observed in the *OEaslA* mutant compared to the WT strain following transfer of liquid cultured mycelia to liquid MMT (Fig. 4C). ST production was similarly decreased by *aslA* overexpression (Fig. 4E). Accordingly, we propose that AslA is an important negative regulator of *aflR* expression at the transcriptional level in both *veA* and *veA1* backgrounds.

To address whether AslA additionally affects the synthesis of secondary metabolites other than ST, we evaluated the effect of deletion and overexpression of *aslA* on expression of the genes involved in TQ biosynthesis, *tdiA* and *tdiB*. During asexual differentiation induced by shifting mycelia from liquid to solid MMG, the mRNA levels of both *tdiA* and *tdiB* were significantly higher in the Δ*aslA* than the WT strain (Fig. 4F). On the other hand, *aslA* overexpression induced by shifting mycelia of the *OEaslA* strain from liquid MMG to liquid MMT resulted in dramatically lower levels of *tdiA* and *tdiB* expression, relative to the WT strain (Fig. 4G). These results suggest that AslA functions as a negative regulator of TQ gene transcription.

### *aslA* is differentially expressed during late growth and early developmental stages, and AslA is localized in nuclei of vegetative hyphae and developmental structures except spores

**Figure 5:**
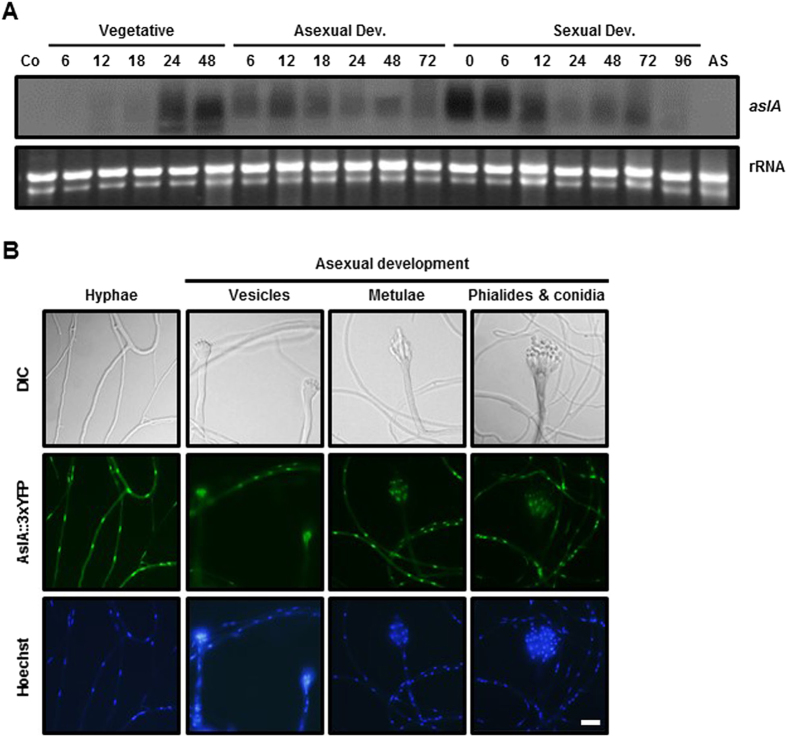
*aslA* is differentially expressed during late growth and early developmental stages, and AslA is localized in the nuclei of mycelia and developmental structures except for spores. (**A**) Northern blot analysis of *aslA* mRNA through the lifecycle of *A. nidulans* FGSC4. Vegetative mycelia were harvested from the culture grown in liquid MMG inoculated with 1.0 × 10^5^ conidia/ml conidia and shake cultured at 120 rpm. Asexual development was induced by shifting the vegetative mycelia grown for 18 h in liquid MMG onto solid MMG followed by incubation under normoxic conditions. For induction of sexual development, the shifted vegetative mycelia were subjected to hypoxia for 24 h followed by incubation under normoxic conditions. Total RNAs were extracted from the vegetative and differentiating mycelia harvested from the cultures after the time intervals indicated. Conidia are indicated as Co, and ascospore as AS. Equal loading of total RNA was confirmed by ethidium bromide staining of rRNA. Full-length gels are presented in Supplementary Figure S4A and B. (**B**) Intracellular localization of AslA-YFP fusion protein. The *C*’*aslA::YFP* strain (MCBA253) was coverslip-cultured on solid MMG for 2–3 days and observed by DIC and fluorescence microscopy. Bar, 20 μm.

To determine the expression profile of *aslA* through the life cycle, Northern blot analysis was performed for total RNAs extracted from spores, vegetative mycelia and developmentally induced cultures of *A. nidulans* FGSC4. As shown in Fig. 5A, *aslA* mRNA accumulation was negligible during the early and middle stages (6, 12 and 18 h) of vegetative growth while considerably high levels were detected during the late stages (24 and 48 h). Moderate to low levels of *aslA* transcript were detected throughout asexual development, with an expression peak at 12 h post-asexual induction. During sexual differentiation, *aslA* transcript was most abundant during the early stages (0–12 h post-sexual induction) and decreased thereafter. No signs of *aslA* transcript was detected in the samples from conidia and ascospores. Thus, we conclude that *aslA* is mainly expressed during the early stages of asexual and sexual development as well as the late vegetative stages, and possibly plays a role during both asexual and sexual development, but not maturation of spores.

The deduced amino acid sequence of AslA comprises 306 amino acids (Mr 35.6 kDa) containing tandem C_2_H_2_ zinc fingers near the *N*-terminus and a Gln-rich domain in the posterior region (Supplementary Figure S6)^36^. Accordingly, we hypothesized that AslA function as a TF and is mainly localized in nuclei. To determine the intracellular localization of AslA, the *C*’*aslA::YFP* strain (MCBA253) expressing YFP-tagged AslA (AslA-YFP) was coverslip-cultured on solid MMG, which supported vegetative mycelial growth and asexual development. After 2–3 days of coverslip culture, YFP fluorescence was observed in the nuclei of vegetative hyphae and most components of conidiophores, *i.e.*, stalks, vesicles, metulae and phialides, but not nuclei of mature conidia (Fig. 5B). Our findings suggest that AslA is present in the majority of vegetative cells as well as developmental structures, except spores, and localized to their nuclei, where it functions as a transcriptional activator.

### The glutamine-rich region of AslA functions as a transcriptional activation domain

**Figure 6:**
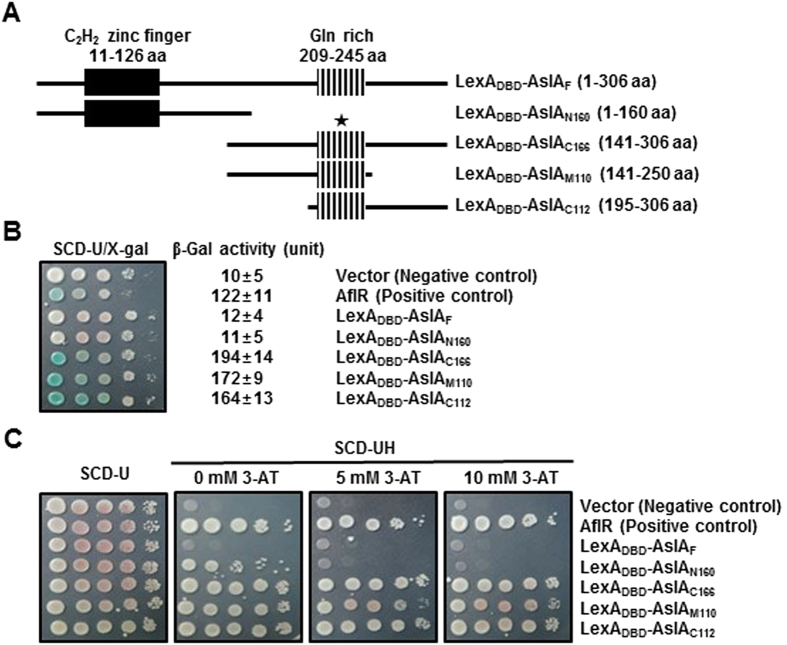
The glutamine-rich region of AslA functions as a transcriptional activation domain. (**A**) Fusion proteins containing various length of AslA partial segments led by LexA_DBD_. Individual PCR amplicons of AslA_F_ (full-length 1-306 aa), AslA_N160_ (1-160 aa), AslA_C167_ (140-306 aa), AslA_M111_ (140-250 aa) and AslA_C107_ (200-306 aa) were cloned in the pTLex vector and fused with LexA_DBD_. A region crucial for transactivation ability of AslA is marked by ★. (**B**) β-Galactosidase reporter assay of transactivation capacity of AslA partial segments. Two-fold serial dilutions of each yeast strain expressing AflR, LexA_DBD_-AslA_F_, LexA_DBD_-AslA_N160_, LexA_DBD_-AslA_C167_, LexA_DBD_-AslA_M111_ and LexA_DBD_-AslA_C107_ were spotted on SCD-U supplemented with X-gal (SCD-U/X-gal), and color of the spots was observed after 2-day culture at 30 °C. These strains were also tested for β-galactosidase activity using ONPG (right). Values are the mean ± SE of five independent experiments. (**C**) His3 reporter assay of transactivation capacity of AslA partial segments. Two-fold serial dilutions of each yeast strain listed in (**B**) were spotted on SCD-U, SCD-UH, SCD-UH/5 mM 3-AT and SCD-UH/10 mM 3-AT, and the plates were incubated for 3 days at 30 °C.

To verify the speculation that AslA acts as a transcriptional activator, we assessed its transactivational capacity and identify the transactivation domain using *β*-galactosidase and His3 reporters in *S. cerevisiae*. First, we constructed yeast transformants of pTLex-derived plasmids that ectopically express fusion proteins containing various lengths of AslA partial segments led by the LexA DNA-binding domain (LexA_DBD_): LexA_DBD_-AslA_F_ (full-length 1–306 aa), LexA_DBD_-AslA_N160_ (1–160 aa), LexA_DBD_-AslA_C167_ (140–306 aa), LexA_DBD_-AslA_M111_ (140–250 aa) and LexA_DBD_-AslA_C107_ (200–306 aa) (Fig. 6A). To assess the ability to activate the β-galactosidase reporter, we observed the colony colors of transformants grown on SCD-U plates containing X-gal. Yeast strains expressing LexA_DBD_-AslA_C167_, LexA_DBD_-AslA_M111_ and LexA_DBD_-AslA_C107_ exhibited blue color within 1 day after inoculation, similar to the strain expressing the Gal4_DBD_-AflR positive control^37^ (Fig. 6B). Data obtained from quantitative analysis of β-galactosidase activity corroborated with transactivation activities of the three fusion proteins. We further evaluated transactivation activity using the His3 reporter. Cells of each transformant were spotted in serial dilution on SCD-UH containing 1 mM or 5 mM 3-AT. As expected, strains expressing one of the three fusion proteins exhibiting positive results in X-gal and β-galactosidase assays were able to grow under these conditions (Fig. 6C). On the other hand, yeast strains expressing LexA_DBD_-AslA_N160_ and LexA_DBD_-AslA_F_ exhibited negligible signs of tansactivation in both *β*-galactosidase and His3 reporter assays (Fig. 6A–C). The results collectively indicate that a transactivation domain centered by a glutamine-rich region (aa 209–245) resides within positions 200–250 of AslA (marked by star in Fig. 6A) and that the N-terminal half is involved in modulatory or inhibitory effects on transactivation. A similar phenomenon has been reported for the developmental regulators, FlbB^20^ and FlbE^23^, in *A. nidulans*.

### AslA may be functionally conserved in aspergilli

**Figure 7:**
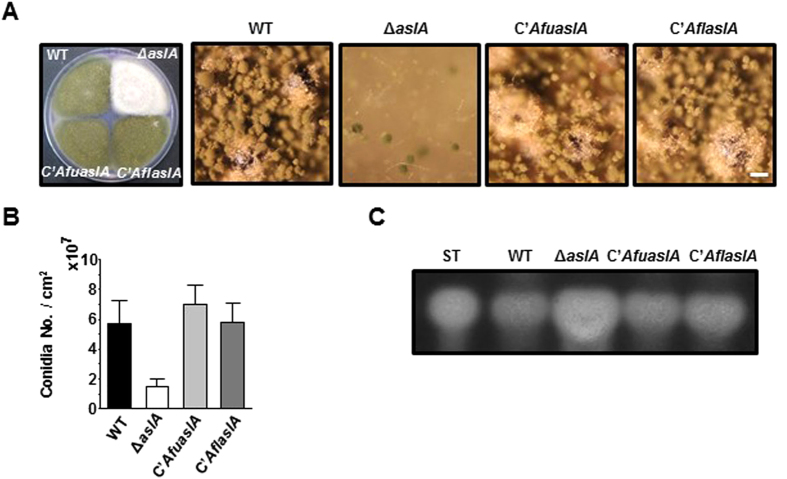
AslA may be functionally conserved in aspergilli. (**A**) Colony morphology of WT (MCBA003), Δ*aslA* (MCBA103), *C*’*AfuaslA* (MCBA605) and *C*’*AflaslA* (MCBA615) strains. Colonies were grown for 4 days after point-inoculation on solid MMG. Entire colonies and close-up views of the center of individual colonies are shown. Bar, 100 μm. (**B**) Quantitative analyses of conidiation by the strains shown in (**A**) performed in triplicate (***P < 0.001). (**C**) TLC analyses of ST in the chloroform extracts of the strains shown in (**A**) grown on plates for 5 days. ST, ST standard. Full-length TLC plates are presented in Supplementary Figure S5.

To determine the functional conservation of AslA among members of the genus *Aspergillus*, we examined whether the *aslA* orthologs from *A. fumigatus (AfuaslA*) and *A. flavus (AflaslA*) complement the phenotype of Δ*aslA* mutation in *A. nidulans*. Introduction of *AfuaslA* and *AflaslA* at the *pyroA* locus yielding C’*AfuaslA* (MCBA605) and C’*AflaslA* (MCBA615) strains fully rescued the defects in asexual differentiation induced by Δ*aslA* mutation (Fig. 7A and B). We additionally determined whether *AfuaslA* and *AflaslA* complement the ST-overproducing phenotype of the Δ*aslA* mutant. Similar to the WT strain, the C’*AfuaslA* and C’*AflaslA* strains showed lower level of ST production compared to the Δ*aslA* strain when grown on solid MMG for 5 days (Fig. 7C). This result indicates that both *AfuaslA* and *AflaslA* negatively regulate ST biosynthesis in *A. nidulans* similar to *aslA*. Accordingly, we propose that the functions of AslA in asexual development as well as secondary metabolism are conserved among the three aspergilli, *A. nidulans, A. fumigatus* and *A. flavus*.

## Discussion

While numerous studies over several decades have focused on the processes of development and accompanying metabolic alterations in the model filamentous fungus, *A. nidulans*, the molecular networks modulating the expression of genes required for asexual differentiation and secondary metabolism remain to be established. In the present study, we characterized the putative C_2_H_2_-type zinc finger TF, AslA, shown to attenuate K^+^ stress-inducible expression of genes encoding vacuolar K^+^ pumps and proteins involved in vacuolar biogenesis^36^, in relation to both asexual differentiation and secondary metabolism.

An initial clue into the regulatory function of AslA in asexual development was obtained from the finding that deletion of *aslA* leads to the formation of fluffy colonies with significantly decreased conidia (Fig. 1A and B), while its overexpression induces a hyper-conidiating phenotype (Fig. 1C–E). The data indicate that AslA plays an important role in asexual differentiation. Deletion and overexpression of *aslA* significantly modulated the mRNA levels of *brlA, abaA* and *wetA* (Fig. 2A and B). In addition, the fluffy phenotype of the Δ*aslA* mutant was suppressed upon *brlA* overexpression (Fig. 2C), leading to the hypothesis that *aslA* is located upstream of *brlA* in the genetic network controlling asexual development and positively regulates the pivotal CDP gene, *brlA*. As described in the introduction, activities of various UDAs, including FluG, FlbA, FlbB, FlbC, FlbD and FlbE, regulate *brlA* expression^3^,^12^,^13^. Three of the UDA members have been identified as TFs closely involved in activation of *brlA* expression, specifically, a bZIP-type TF, FlbB^21^,^22^,^38^, C_2_H_2_ zinc-finger TF, FlbC^19^, and cMyb-type TF, FlbD^21^. Thus AslA is probably a novel TF belonging to the UDA members. However, further studies are essential to determine the hierarchical relationship between AslA and other UDA members.

In contrast to members of the FluG-initiated UDA network, the two key activators of sexual reproduction, NsdD and VeA, play negative roles in asexual development by suppressing *brlA* expression^24^,^25^,^27^. Analysis of the phenotypes of the double mutants, Δ*aslA* Δ*nsdD* and Δ*aslA veA1*, revealed that either *nsdD* deletion or *veA1* mutation suppresses the impaired conidiation phenotype triggered by *aslA* deletion (Fig. 3A and B). Accordingly, either nullifying *nsdD* or introducing *veA1* mutation in the Δ*aslA* mutant at least partially restored the expression of *brlA* (Fig. 3C). Thus, both NsdD and VeA are suggested to function downstream of AslA to control *brlA* expression. In agreement with these results, deletion of *nsdD* suppressed all developmental defects caused by null mutations of the UDA genes, including *fluG, flbA, flbB, flbC* and *flbE*, but not Δ*brlA*^24^. Although limited information is available on suppression of the *flb* mutations by *veA1* or Δ*veA* mutations, similar results are expected as for *nsdD*.

We investigated whether AslA participates in the regulation of secondary metabolite production by analyzing the effects of deletion and overexpression of *aslA* on expression of ST biosynthetic genes. Our data indicate that AslA acts as a negative regulator of ST biosynthesis by suppressing the expression of *aflR* and *stcU* encoding a Zn(II)_2_Cys_6_ TF and ST biosynthetic enzyme, respectively (Fig. 4A–E). Additionally, AslA negatively controls the expression of TQ biosynthetic genes, *tdiA* and *tdiB* (Figs 4F and 6G). It has long been accepted that in filamentous fungi, the regulatory gene networks for secondary metabolism and morphological development are intimately associated via a considerable number of common regulators^26^,^39^,^40^. For example, the putative C_2_H_2_ zinc finger TF, MtfA, positively regulates conidiophore formation via activation of *brlA* expression as well as ST production via effects on *aflR* expression^41^. Interestingly, deletion of *mtfA* or its overexpression leads to reduction of *aflR* transcription, suggesting that only wild-type MtfA levels in balanced stoichiometry with other relevant factors are required for normal ST production. MtfA is also a positive regulator of other secondary metabolism gene clusters, including those responsible for TQ biosynthesis. Additionally, LaeA, the first methyltransferase discovered to associate with VeA, is not only required for activation of *aflR* expression but also involved in the transcription of gene clusters for other secondary metabolites^29^,^30^. In the absence of light, LaeA associates with the VelB-VeA dimer to form the heterotrimeric velvet complex, VelB-VeA-LaeA, which performs chromatin remodeling required for expression of *aflR* and other secondary metabolite biosynthesis genes^28^,^31^. LaeA also plays an important role in light-dependent conidia production, which requires the presence of intact VeA protein^42^. In contrast to MtfA and LaeA that play positive roles in both asexual differentiation and ST biosynthesis, AslA provides a prime example of a TF that positively controls asexual differentiation by activating *brlA* expression, but negatively affects ST biosynthesis by suppression of *aflR* expression. Correspondingly, in spite of its significant defects in asexual development, Δ*aslA* mutant showed increased production of ST (Figs 1 and 4). On the basis of the data presented here, we propose that AslA is a novel regulator that may act at the split control point of the developmental and metabolic pathways. However, for clear understanding of the branch point, it should be rigorously established whether AslA modulates the expression *aflR* and *brlA* directly or indirectly through unknown regulator(s).

On the basis of the deduced amino acid sequence of AslA containing tandem C_2_H_2_ zinc fingers near the *N*-terminus and a Gln-rich domain in the posterior portion (see Supplementary Figure S6A)^36^, we attempted to determine whether this protein meets the basic criteria for functioning as a TF, *i.e.* nuclear localization and transcriptional activation. The AslA-YFP fusion protein predominantly accumulated in nuclei of not only vegetative cells but also differentiating cells in conidiophores, vesicles, metulae and phialides, except conidia (Fig. 5B). However, we found no evidence of differential localization of AslA according to developmental status of the fungal cells or environmental conditions. Using *β*-galactosidase and His3 reporters in *S. cerevisiae*, we showed that AslA has transactivation ability, which is mediated by a putative transactivation domain (200–250 aa) centered by a glutamine-rich region (Fig. 6). Generally, transcriptional activation functions of TFs are based within glutamine-rich, acidic, or proline-rich domains localized outside DNA-binding regions, such as C_2_H_2_, Zn(II)_2_Cys_6_ and GATA zinc fingers^43^. Transactivational function of the glutamine-rich domain is conserved among a wide variety of eukaryotes, from yeast to human^44^. In the filamentous fungus *Neurospora crassa*, Nit4, a single Zn(II)_2_Cys_6_ binuclear-type zinc finger protein, contains a glutamine-rich domain that functions in transcriptional activation^45^. The transactivation domains of several developmental regulators, such as FlbB^20^, FlbC^19^ and FlbE^23^, have been mapped in *A. nidulans*, none of which harbor a glutamine-rich domain with transactivation capacity. Thus, AslA appears to provide a primary example for a developmental regulator carrying a glutamine-rich transactivation module.

Previous reports have shown that proteins sharing significant sequence similarity with AslA are mainly present in members of the family Trichocomaceae, including the genera *Aspergillus, Penicillium* and *Talaromyces* (see Supplementary Figure S6B)^36^. Results from the present study indicate that the *aslA* orthologs from *A. fumigatus (AfuaslA*) and *A. flavus (AflaslA*) complement the Δ*aslA* phenotype related to asexual differentiation and ST production in *A. nidulans* (Fig. 7). Accordingly, the role of AslA in development and secondary metabolism seems to be conserved across the members of the genus *Aspergillus*.

Based on our findings in the present study, we propose that AslA is a novel regulator that oppositely regulates development and ST biosynthesis at the split control point of the developmental and metabolic pathways. Further studies on the hierarchical relationship between *aslA* and other UDA gens would contribute to expand our understanding of the regulatory networks governing the processes of fungal development and secondary metabolite production.

## Methods

### Strains, media and cultivation

*A. nidulans* strains used in this study are listed in Table 1. MMG was prepared as described previously^36^,^46^. For overexpression of selected genes driven by the promoter of *alcA* gene (*alcA(p*)), MMT which was identical to MMG except that it contained 0.5% yeast extract and 100 mM threonine instead of 2% glucose was used. For preparation of solid media, 1.5% agar was added to the liquid media. Unless otherwise indicated, cultures for all experiments described were grown at 37 °C.

To prepare vegetative mycelia, conidia of *A. nidulans* strains were inoculated into liquid MMG to a concentration of 1.0 × 10^5^ conidia/ml and grown in a shaking culture at 120 rpm. Mycelia were then harvested by filtering the culture through a Miracloth filter (Calbiochem, USA). To induce synchronized asexual differentiation, vegetative mycelia grown for 18 h in liquid MMG were spread onto solid MMG and incubated under air-exposed conditions. The whole cells undergoing asexual differentiation were harvested from the plates at designated time points after transfer. For microscopic observation of the fungal hyphae, each *A. nidulans* strain was coverslip-cultured on a block of appropriate agar medium under a coverslip on a glass slide for several days. For plasmid amplification, *Escherichia coli* DH5α was cultured in Luria-Bertani medium supplemented with 50 μg/ml of ampicillin (Sigma-Aldrich, USA).

### Molecular techniques

Standard DNA transformation procedures were used for *A. nidulans*^47^ and *E. coli*^48^. For PCR experiments, standard protocols were applied using a MyGenie 96/384 Gradient Thermal Block (Bioneer, Korea) for reaction cycles. Genomic DNA was extracted from the spores or mycelia of *A. nidulans* as described previously^49^,^50^. Primers for PCR are listed in Supplementary Table S1.

Total RNA was isolated with Trizol according to the manufacturer’s protocols (Invitrogen, USA). Northern hybridization was performed using standard techniques^48^. *aslA* probe for Northern blots were amplified by PCR from *A. nidulans* genomic DNA with the primers, PaslA-Nf and PaslA-Nr. For RT*-*qPCR, first strand cDNA was copied from a total RNA preparation using M-MLV reverse transcriptase (Enzynomics, Korea) according to the manufacturer’s protocol. RT-qPCR was performed using a Bio-Rad CFX96 Real-Time PCR System (Bio-Rad, USA) and a TOPreal™ qPCR 2X PreMIX Kit (Enzynomics) with the primers against the genes of interest and 18S rRNA (internal control) (see Supplementary Table S1). Expression levels of target genes versus the 18S rRNA gene were computed using the 2^−ΔCt^ method described previously^36^.

### Generation of fungal strains

The green-spored Δ*aslA* strains, MCBA103 (*pyroA4 argB2* Δ*aslA::argB*) and MCBA104 (*pyroA4* Δ*aslA::argB*; *veA1*), were generated by a sexual cross between MCBA101 (*yA2 pyroA4* Δ*aslA::argB*) and FGSC26 (*biA1 veA1*) strains according to standard methods^46^. The reference strains, MCBA003 (*pyroA4*) and MCBA004 (*pyroA4 veA1*), were constructed by crossing TJ1 (*yA2 pyroA4*) with FGSC26.

For complementation of Δ*aslA* mutation with a hybrid gene encoding FLAG-tagged AslA, a 4.1-kb *aslA(p)::aslA*_*orf*_ fragment containing the presumptive promoter *aslA(p*) (3.0 kb) and *aslA*_*orf*_ (1.1-kb) was amplified from the genomic DNA of FGSC4 by PCR using the primers, PaslA4-f and PaslA4-r, and cloned into the TOPcloner (Enzynomics) to yield TOP-aslA4. The 4.1-kb *aslA(p)::aslA*_*orf*_ fragment was excised from TOP-aslA4 by *Kpn*I-*Hin*dIII digestion and cloned into *Kpn*I-*Hin*dIII-digested pHS13^51^ to yield pHS-aslA-FLAG. The *aslA* complemented (*C*’*aslA*) strain, MCBA203 (*pyroA::aslA(p)::aslA::FLAG*_*3x*_*::trpC(t)::pyroA* Δ*aslA::argB*), was generated by transformation of the *aslA*-null strain (MCBA103) with pHS-aslA-FLAG plasmid. A hybrid gene encoding YFP-tagged AslA fusion protein was constructed as follows. First, a 2.3-kb 3 × *YFP* fragment was excised from pBS-3 × YFP^52^ by *Eco*RI digestion and cloned into pHS13 to yield pHS-YFP. The 4.1-kb *aslA(p)::aslA*_*orf*_ fragment was similarly PCR amplified with the primers, PC’YaslA-4f and PC’YaslA-4r, and cloned into *Pvu*II-cut pHS-YFP to yield pHS-aslA-YFP. The *aslA*-null strain (MCBA103) was then transformed with pHS-aslA-YFP to yield the C’aslA::YFP strain, MCBA253 (*pyroA::aslA(p)::aslA::YFP3x::FLAG3x trpC(t)::pyroA* Δ*aslA::argB*).

For construction of *aslA-*overexpressing (*OEaslA*) strains, the *aslA*_*orf*_ was amplified from the genomic DNA of FGSC4 using the primers, POEaslA-1f and POEaslA-1r. The resulting 1.1-kb *aslA*_*orf*_ fragment was cloned into TOPcloner to yield TOP-aslA_ORF_. The 1.1-kb *Hin*dIII fragment of *aslA*_*orf*_ was excised and cloned into *Hin*dIII-digested pHS3^51^ downstream of *alcA(p*) to yield pHS-alcA(p)-aslA-FLAG. The final recombinant plasmids were used to transform the reference strain, MCBA003, to yield the *OEaslA* strain, MCBA303 (*pyroA::alcA(p)::aslA::FLAG::trpC(t)::pyroA*).

To generate *brlA-*overexpressing strains, the *brlA*_*orf*_ was amplified from the genomic DNA of FGSC4 using the primers, POEbrlA-1f and POEbrlA-1r. The resulting 1.3-kb *brlA*_*orf*_ fragment was cloned into TOPcloner to yield TOP-brlA_ORF_. The 1.6-kb *Bam*HI-*Not*I fragment of *brlA*_*orf*_ was excised and cloned into *Bam*HI-*Not*I-digested pHS3 downstream of *alcA(p*) to yield pHS-alcA(p)-brlA-FLAG. The recombinant plasmid was introduced into the reference (MCBA003) and Δ*aslA* (MCBA103) strains to yield *OEbrlA* strain, MCBA353 (*pyroA::alcA(p)::brlA::FLAG::trpC(t)::pyroA*), and Δ*aslA OEbrlA* strain, MCBA553 (*pyroA:: alcA(p)::brlA::FLAG::trpC(t)::pyroA* Δ*aslA::argB*), respectively. The Δ*aslA* Δ*nsdD* double mutant strains, MCBA403 (Δ*aslA::argB* Δ*nsdD::AfupyrG*) and MCBA404 (Δ*aslA::argB* Δ*nsdD::AfupyrG veA1*), were generated by a cross between MCBA101 and TNJ111 (Δ*nsdD::AfupyrG veA1*) strains.

For complementation of Δ*aslA* mutation by the *aslA* orthologues from *A. fumigatus (AfuaslA*) and *A. flavus (AflaslA*), 5.0-kb DNA fragments of *AfuaslA* and *AflaslA* loci were amplified by PCR from the genomic DNAs of *A. fumigatus* (AF293) with the primers, PC’AfuaslA-f and PC’AfuaslA-r, and *A. flavus* (NRRL3375) with the primers, PC’AflaslA-f and PC’AflaslA-r, respectively. The resulting amplicons were cloned individually into TOPcloner to yield TOP-AfuaslA and TOP-AflaslA. The 5.0-kb *Hin*dIII-*Not*I fragment of *AfuaslA* gene excised from TOP-AfuaslA was cloned into *Hin*dIII-*Not*I-digested and pHS7^23^ to yield pHS-AfuaslA. Similarly, the 5.0-kb *Bam*HI-*Not*I fragment of *AflaslA* gene excised from TOP-AflaslA was cloned into *Bam*HI-*Not*I-digested pHS7 to yield pHS-AflaslA. The *aslA*-null strain (MCBA103) was then transformed with pHS-AfuaslA and pHS-AflaslA to yield the *C*’*AfuaslA* and *C*’*AflaslA* strains, MCBA605 (Δ*aslA::argB AfuaslA::pyroA*) and MCBA615 (Δ*aslA::argB AflaslA::pyroA*), respectively.

### Sterigmatocystin analysis

ST was extracted with chloroform from the mycelia of *A. nidulans* strains harvested from solid or liquid cultures^24^. Briefly, 0.2 g (wet weight) of mycelium was mixed 10 ml of chloroform and incubated for 30 min at room temperature with vigorous vortexing in about 5-min intervals. The organic phase (lower) was harvested and centrifuged (700× g, 5 min). The resulting chloroform layer was collected, dried and resuspended in 100 μl of chloroform. Approximately 20 μl of each sample and ST standard (5 mg; Sigma-Aldrich) were loaded onto a TLC silica plate (Silica gel 60 F254; Merck, Germany). The plate was then developed in a mobile phase composed of toluene:ethylacetate:acetic acid (80:10:10, v/v/v), and ST spots were visualized by spraying aluminum chloride (20% w/v in 95% ethanol) on the TLC plate followed by baking the plate at 70 °C for 5 min. Photographs of TLC plates were taken following exposure to UV of 320 nm.

### Microscopy

Coverslip culture was performed as described previously^36^. The coverslips were stained with 1 mg/ml Hoechst 33342 (Sigma) for 10 min, briefly washed with distilled water and dipped in distilled water for 10 minutes. Then the coverslips were washed with ethanol, air-dried for 5 minutes and mounted with antifade mounting medium (H-1000; Vectashield, USA). For differential interference contrast (DIC) and fluorescence microscopy, an Olympus System microscope Model BX51 (Olympus, Japan) equipped with UPlanSApo 60× and UPlanFL 100× objective lenses (Olympus) were used. DAPI (High brightness) filter cube (Excitation filter: center wavelength 377 nm, Emission filter: center wavelength 447 nm; Olympus) and FITC filter cube (Excitation filter: center wavelength 483 nm, Emission filter: center wavelength 535 nm; Olympus) were used to observe the fluorescence of Hoechst and YFP, respectively. Images were captured with a DP71 digital camera (Olympus) and processed using the DP manager imaging software (Olympus) and Photoshop CS5.1 (Adobe Systems, USA).

### Mapping a transactivation domain in AslA

The transactivating capacity of AslA was determined by using a modified yeast one hybrid system^19^. Briefly, cDNA fragments encoding the full-length and various partial segment of AslA were amplified by PCR from *aslA* cDNA using the primers as follows: AslA_F_ (full-length 1–306 aa; PaslA-1f and PaslA-306r), AslA_N160_ (1–160 aa; PaslA-1f and PaslA-160r), AslA_C166_ (141–306 aa; PaslA-141f and PaslA-306r), AslA_M110_ (141–250 aa; PaslA-141f and PaslA-250r) and AslA_C112_ (195–306 aa; PaslA-195f and PaslA-306r). After *Sma*I-*Xho*I digestion, the amplicons were individually fused with the coding sequence of LexA DNA-binding domain (LexA_DBD_) in the pTLex vector (kindly provided by Suhn-Kee Chae, Paichai University, Daejeon, Korea) to yield pLexA_DBD_-AslA_F_, pLexA_DBD_-AslA_N160_, pLexA_DBD_-AslA_C166_, pLexA_DBD_-AslA_M110_ and pLeA_DBD_x-AslA_C112_. The resulting plasmids were individually introduced into *Saccharomyces cerevisiae* L40^53^. For X-gal plate assay for visualization of *β*-galactosidase expression mediated by the LexA_DBD_ fusion proteins, two-fold serial dilutions of each transformants were spotted on synthetic complete dextrose (SCD) medium lacking uracil (SCD-U)^54^ supplemented with 40 mg/ml 5-bromo-4-chloro-3-indolyl-*β*-D-galactopyranoside (X-gal; Sigma-Aldrich), and color of the spots was observed after 2-day culture at 30 °C. For quantitative analysis *β*-galactosidase expression, the yeast transformants were tested for *β*-galactosidase activity using a yeast *β*-galactosidase assay kit that contained the substrate *o*-nitrophenyl-*β*-D-galactopyranoside (ONPG; Sigma-Aldrich). For His3 reporter assay, we dilution-spotted the cells of each transformant on SCD lacking uracil and histidine (SCD-UH) supplemented with 5 mM 3-amino-1,2,4-triazole (3-AT; Sigma-Aldrich) and evaluated for their growth after 2-day incubation at 30 °C.

## Additional Information

**How to cite this article**: Kim, Y. J. *et al*. Differential control of asexual development and sterigmatocystin biosynthesis by a novel regulator in *Aspergillus nidulans. Sci. Rep.* **7**, 46340; doi: 10.1038/srep46340 (2017).

**Publisher's note:** Springer Nature remains neutral with regard to jurisdictional claims in published maps and institutional affiliations.

## Supplementary Material

Supplementary Information Files

## Figures and Tables

**Table 1:** *Aspergillus* strains used in this study.

Strain	Relevant genotype	Source/Reference
*A. nidulans*		
FGSC4	*veA*^+^	FGSC^a^
FGSC26	*biA1*; *veA1*	FGSC^a^
TNJ108	*pyrG89*; *pyroA4*; Δ*nsdD::AfupyrG*; *veA*^+^	24
TNJ111	*pyrG89*; *pyroA4*; Δ*nsdD::AfupyrG*; *veA1*	24
MCBA003	*pyroA4*; *veA*^+^	This study
MCBA004	*pyroA4*; *veA1*	This study
MCBA101	*yA2*; *argB2*; *pyroA4*; Δ*aslA::argB*; *veA*^+^	36
MCBA103	*argB2*; *pyroA4*; Δ*aslA::argB*; *veA*^+^	This study
MCBA104	*argB2*; *pyroA4*; Δ*aslA::argB*; *veA1*	This study
MCBA203	*argB2*; *pyroA4, pyroA::aslA::FLAG*_*3x*_*::trpC(t)::pyroA*^b^; Δ*aslA::argB*; *veA*^+^	This study
MCBA253	*argB2*; *pyroA4, pyroA::aslA::YFP*_*3x*_*::FLAG*_*3x*_*::trpC(t)::pyroA*^b^; Δ*aslA::argB*; *veA*^+^	This study
MCBA303	*pyroA4, pyroA::alcA(p)::aslA::FLAG::trpC(t)::pyroA*^b^; *veA*^+^	This study
MCBA353	*pyroA4, pyroA::alcA(p)::brlA::FLAG::trpC(t)::pyroA*^b^; *veA*^+^	This study
MCBA403	*pyrG89*; *argB2*; Δ*aslA::argB*; Δ*nsdD::AfupyrG*; *veA*^+^	This study
MCBA404	*pyrG89*; *argB2*; Δ*aslA::argB*; Δ*nsdD::AfupyrG*; *veA1*	This study
MCBA553	*argB2*; *pyroA4, pyroA::alcA(p)::brlA::FLAG::trpC(t)::pyroA*^b^; Δ*aslA::argB*; *veA*^+^	This study
MCBA605	*argB2*; *pyroA4, pyroA::AfuaslA::pyroA*^b^; Δ*aslA::argB*; *veA*^+^	This study
MCBA615	*argB2*; *pyroA4, pyroA::AflaslA::pyroA*^b^; Δ*aslA::argB*; *veA*^+^	This study
*A. fumigatus*		
AF293		55
*A. flavus*		
NRRL 3375		56

^a^Fungal Genetics Stock Center (Kansas City, KN, USA).

^b^The 3/4 *pyroA* marker causes the targeted integration at the *pyroA* locus.
