# Erratum: Differential Control of Asexual Development and Sterigmatocystin Biosynthesis by a Novel Regulator in *Aspergillus nidulans*

**DOI:** 10.1038/srep46817

**Published:** 2017-05-26

**Authors:** Yong Jin Kim, Yeong Man Yu, Pil Jae Maeng

Scientific Reports 7: Article number: 4634010.1038/srep46340; published online: 04 19 2017; updated: 05 26 2017

The original version of this Article contained a typographical error in the spelling of the author Yeong Man Yu, which was incorrectly given as Yu Yeong Man. This has now been corrected in both the PDF and HTML versions of the Article.

