# Supplementary material for: Differential Control of Asexual Development and Sterigmatocystin Biosynthesis by a Novel Regulator in Aspergillus nidulans
Source: Sci Rep. 2017 Apr 19;7:46340. doi: 10.1038/srep46340 (PMC5396049; doi:10.1038/srep46340)
Supplement: Supplementary Information Files [file srep46340-s1.pdf]

Supplementary Information

Differential Control of Asexual Development and Sterigmatocystin Biosynthesis by a Novel Regulator in *Aspergillus nidulans*

Yong Jin Kim, Yeong Man Yu & Pil Jae Maeng*

Department of Microbiology and Molecular Biology, Chungnam National University, Daejeon 34134, Korea

***Corresponding Author:**

Pil Jae Maeng
Department of Microbiology and Molecular Biology
Chungnam National University
Daejeon 34134, Korea
Tel: +82-42-821-6415
Fax: +82-42-822-7367
E-mail: pjmaeng@cnu.ac.kr

Supplementary Table S1. List of oligonucleotide primers used in this study

Primer	Sequence (5'–3')	Gene (locus)
For Northern blot probe		
PaslA-Nf	TCCTCAAGTCTTGCTCGCCA	*aslA*
PaslA-Nr	AAGGGTTGAGGCATAGGGCA	*aslA*
For Southern blot probe		
PbrlA-Sf	ATGCGAAATCAGTCCAGCCTGTCC	*brlA*
PbrlA-Sr	TGGCAACATGTTATTCATTGACAT	*brlA*
For deletion, complementation and overexpression strains		
PC'aslA-4f	GGTACCGAGCTGACGCTGATCCTTCGAC[b]	*aslA*
PC'aslA-4r	AAGCTTGCACCCAGTTCCAAGGAACGC[b]	*aslA*
PC'YaslA-4f	GAGCTGACGCTGATCCTTCGAC	*aslA*
PC'YaslA-4r	GCACCCAGTTCCAAGGAACGC	*aslA*
PC'AfuaslA-f	AAGCTTCTAACAAGCCACCATTCTCCATTGAC[b]	*AfuaslA*
PC'AfuaslA-r	GCGGCCGCCTAGTTATCTCCACATAGATTCTAGGC[b]	*AfuaslA*
PC'AflaslA-f	GGATCCCCCTCTCCAAATAATCTTGAGCGG[b]	*AflaslA*
PC'AflaslA-r	GCGGCCGCTTACTTTGGTTTCTTCTCTTGAGTACAGA[b]	*AflaslA*
POEaslA-1f	AAGCTTATGGCTCCTGGCAGCGGC[b]	*OEaslA*
POEaslA-1r	AAGCTTGCACCCAGTTCCAAGGAACGC[b]	*OEaslA*
POEbrlA-1f	GGATCCATGCGAAATCAGTCCAGCCTGTC[b]	*OEbrlA*
POEbrlA-1r	GCGGCCGCTTCATCCCAGCCGTCCAGG[b]	*OEbrlA*
For AslA activation domain mapping		
PaslA-1f	CCCGGGATGGCTCCTGGCAGCGG	*aslA*
PaslA-306r	CTCGAGGCACCCAGTTCCAAGGAACGC	*aslA*
PaslA-160r	CTCGAGTAAGAGATACTGCTCGTTCGGATGAG	*aslA*
PaslA-141f	CCCGGGTCGGAACACTCATACTCTACACCTG	*aslA*
PaslA-250r	CTCGAGGTGTGGATCAAAGCCCTGGTTC	*aslA*
PaslA-195f	CCCGGGATCATCCATAGTAATATTCCCGTAACCTC	*aslA*
For RT-qPCR		
P18S-rRNA-qf	CTTGGATTTGCTGAAGACTAACTACTG	18S rRNA

P18S-rRNA-qr	CTAACTTTCGTTCCCTGATTAATGAAAACAT	18S rRNA
PaslA-qf	CCTATGCCTCAACCCTTGAATACAAG	*aslA*
PaslA-qr	AAATCTGTTCCCTCTGGTTGATTTAACAGT	*aslA*
PbrlA-qf	TCATATACAGACTTTCCGACCTCTC	*brlA*
PbrlA-qr	AAGACCTGATTGGGGTAGTGGG	*brlA*
PabaA-qf	AATTGATCTTCCTCCTCTATCCTTAGA	*abaA*
PabaA-qr	TGCCTGGTATAAGTTCGAAGTAGG	*abaA*
PwetA-qf	AACCACGGAGACTTCCTGCAAG	*wetA*
PwetA-qr	ATGAAGATATATCGAATTGTGTAGGGTCTA	*wetA*
PaflR-qf	AGAGCAACCCCGGCAAACTG	*aflR*
PaflR-qr	GCTGTGTCCAAGATTTGTTTGTTGTC	*aflR*
PstcU-qf	TTGAGCACTTCGGATACCTGGATAT	*stcU*
PstcU-qr	TCCTTGACATGCCCGAACGAG	*stcU*
PtdiA-qf	ATTACACCATGTTGAATCGGGAGTATG	*tdiA*
PtdiA-qr	CGCTCCCTCAGTACATTCTTCAAT	*tdiA*
PtdiB-qf	TACACGATTGAGATTACCCCCGG	*tdiB*
PtdiB-qr	TGTACTGGAATAACGGGACGTAGA	*tdiB*

[a] Lowercase letters indicate *pyroA* sequence.
[b] Underlined letters indicate restriction site.

Supplementary Figure S1

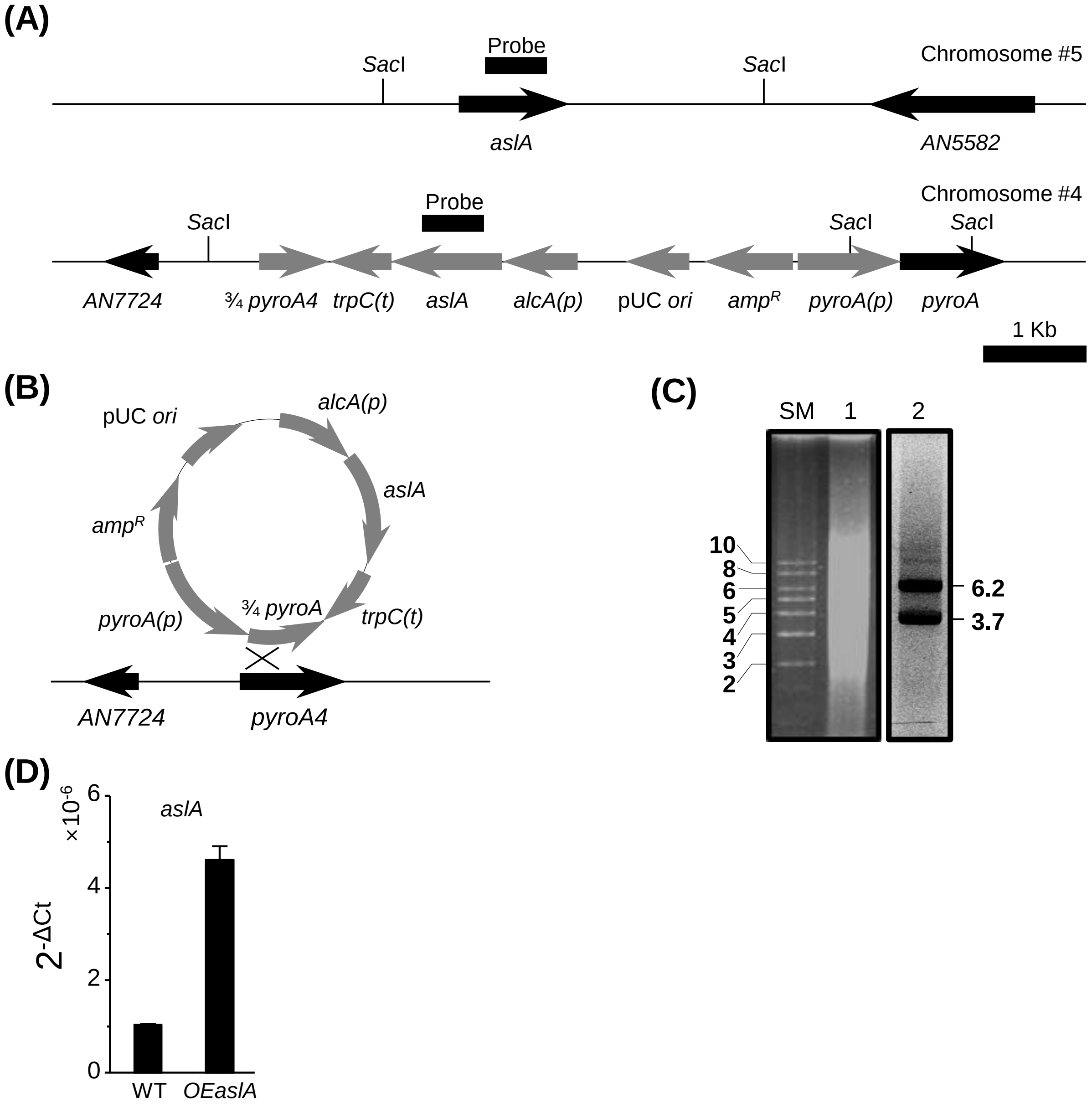

Supplementary Fig. S1. Southern blot and RT-qPCR analyses of the *OEaslA* strain.
(A) Restriction maps of the *aslA* and *pyroA* loci of the *OEaslA* (MCBA353) strain predicted on the basis of the results of Southern blot analysis. Gray arrows represent the essential components of the overexpression vector, pHS-alcA(p)-aslA-FLAG, and dark ones in the chromosomes of the *OEaslA* strain. (B) The mode of pHS-alcA(p)-aslA-FLAG integration at the *pyroA* loci of the *OEaslA* strain. (C) Southern blot analysis of the *OEaslA* strain. Genomic DNA from the *OEaslA* strain was digested with *Sac*I (lane 1), and hybridized with the probe, 0.6-kb *aslA* PCR fragment lane 2). (D) RT-qPCR analysis of *aslA* expression in the WT (MCBA003) and *OEaslA* strains performed in triplicate. Mycelia of the strains grown in liquid MMG for 18 h were shifted to liquid MMT, and the total RNAs were extracted after 12 hr. Primers used for RT-qPCR: *aslA*, PaslA-qf and PaslA-qr; 18S rRNA (internal control), P18S-rRNA-qf and P18S-rRNA-qr.

Supplementary Figure S2

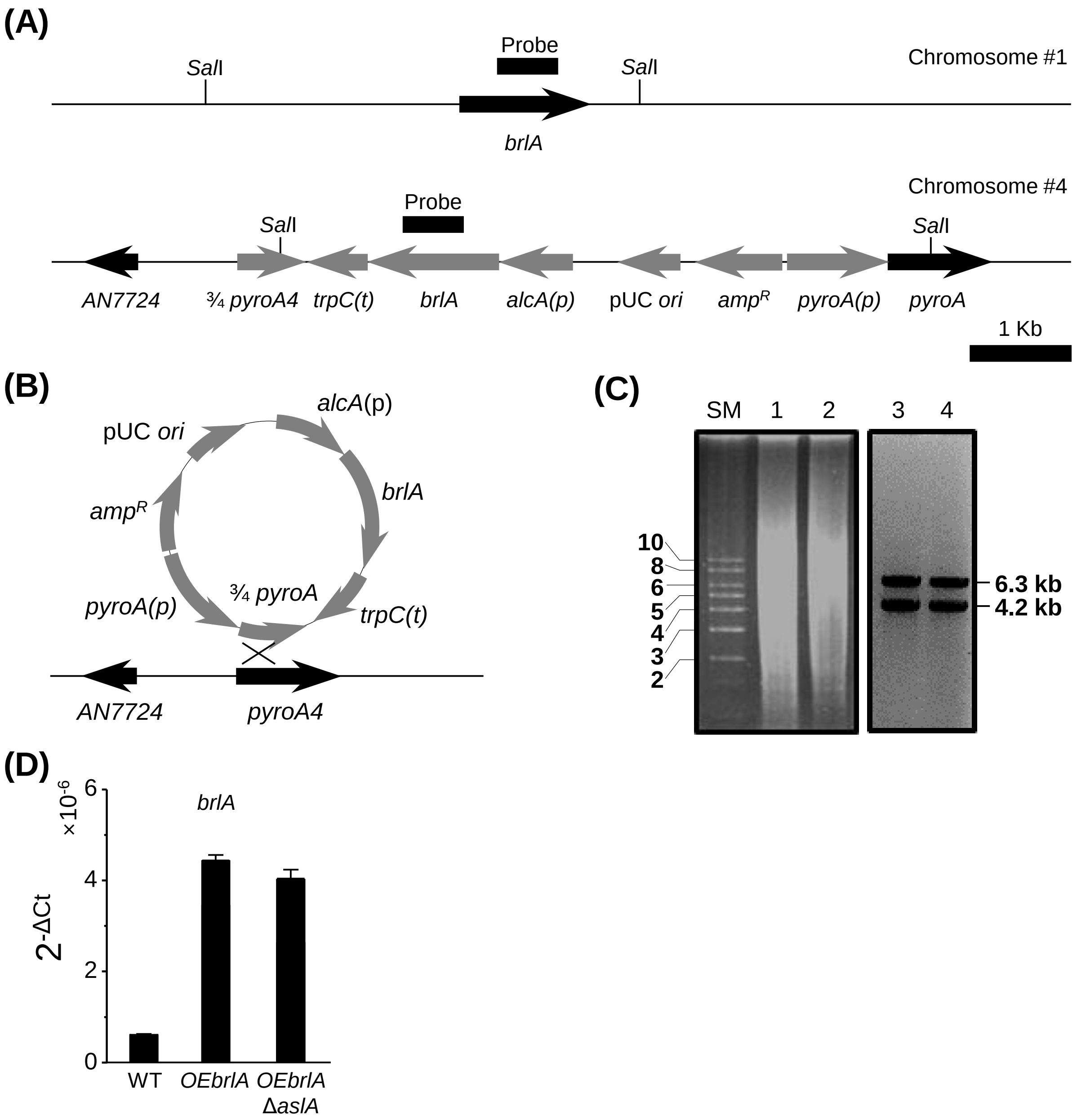

Supplementary Fig. S2. Southern blot and RT-qPCR analyses of the *OEbrlA* and *OEbrlA* Δ*aslA* strains.
(A) Restriction maps of the *brlA* and *pyroA* loci of the *OEbrlA* (MCBA353) and *OEbrlA* Δ*aslA* (MCBA553) strains predicted on the basis of the results of Southern blot analysis. Gray arrows represent the essential components of the overexpression vector, pHS-alcA(p)-brlA-FLAG, and dark ones in the chromosomes of the *OEbrlA* and *OEbrlA* Δ*aslA* strains. (B) The mode of pHS-alcA(p)-brlA-FLAG integration at the *pyroA* loci of the *OEbrlA* and *OEbrlA* Δ*aslA* strains. (C) Southern blot analysis of the OE*brlA* and OE*brlA* Δ*aslA* strains. Genomic DNAs from the *OEbrlA* and *OEbrlA* Δ*aslA* strains were digested with *Sal*I (lane 1-2), and hybridized with the probe, 0.6-kb *brlA* PCR fragment (lane 3-4). (D) RT-qPCR analysis of *brlA* expression in the WT (MCBA003), *OEbrlA* and *OEbrlA* Δ*aslA* strains performed in triplicate. Mycelia of the strains grown in liquid MMG for 18 h were shifted to liquid MMT, and the total RNAs were extracted after 12 hr. Primers used for RT-qPCR: *brlA*, PbrlA-qf and PbrlA-qr; 18S rRNA (internal control), P18S-rRNA-qf and P18S-rRNA-qr.

Supplementary Figure S3

(A)

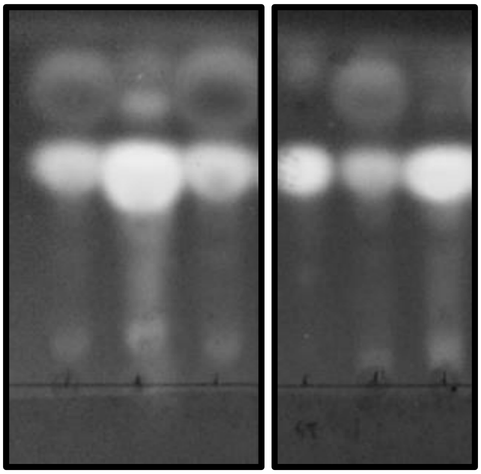

(B)

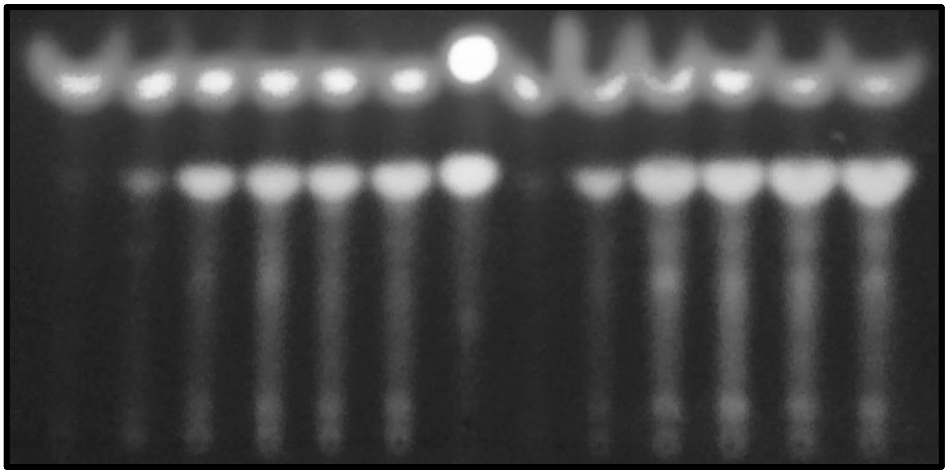

(C)

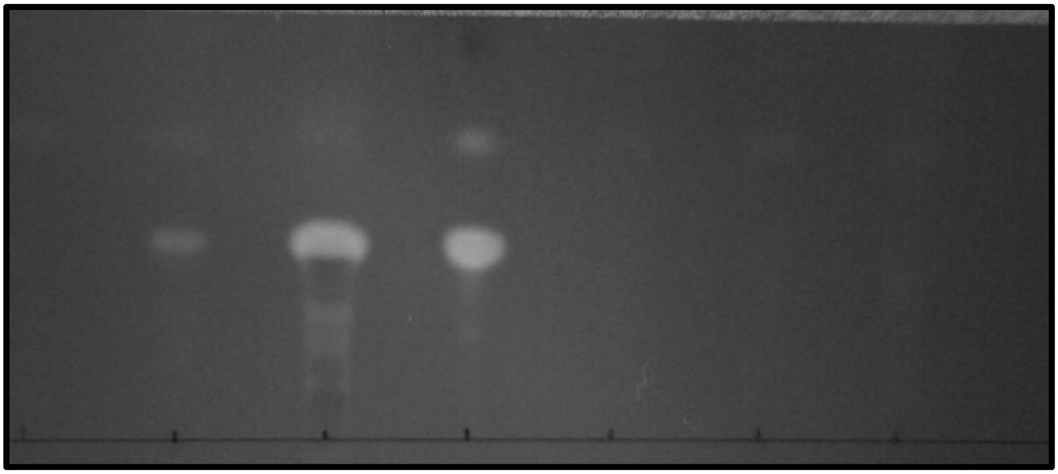

Supplementary Figure S4

(A)

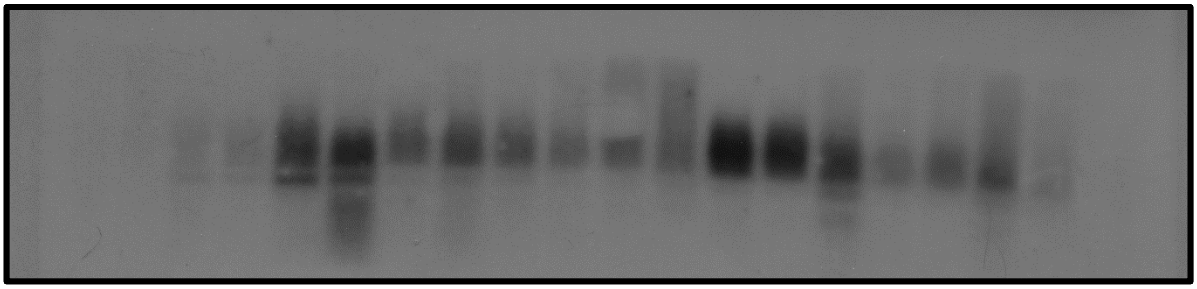

(B)

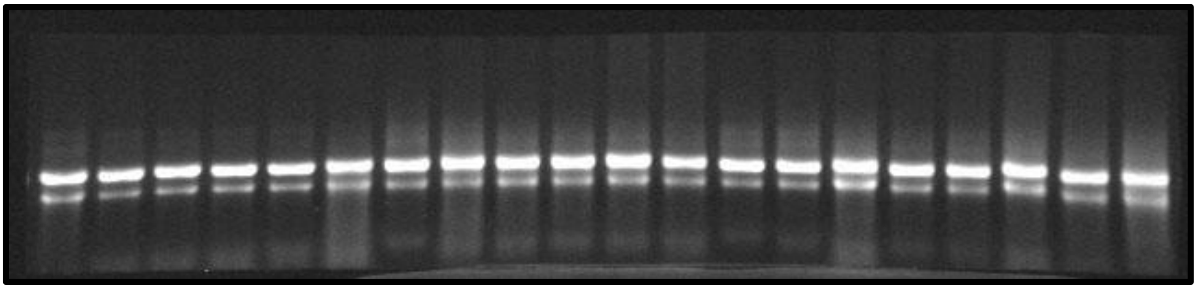

Supplementary Figure S5

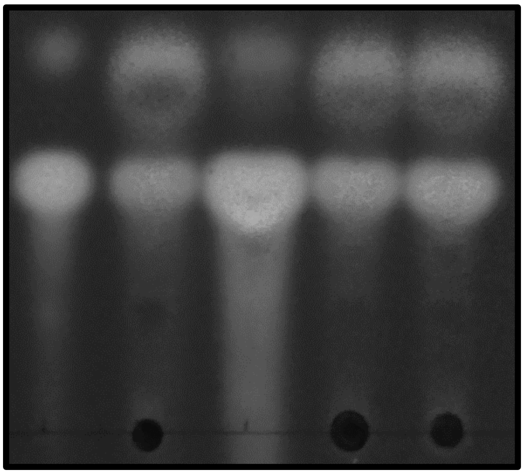

Supplementary Figure S6[36]

(A)

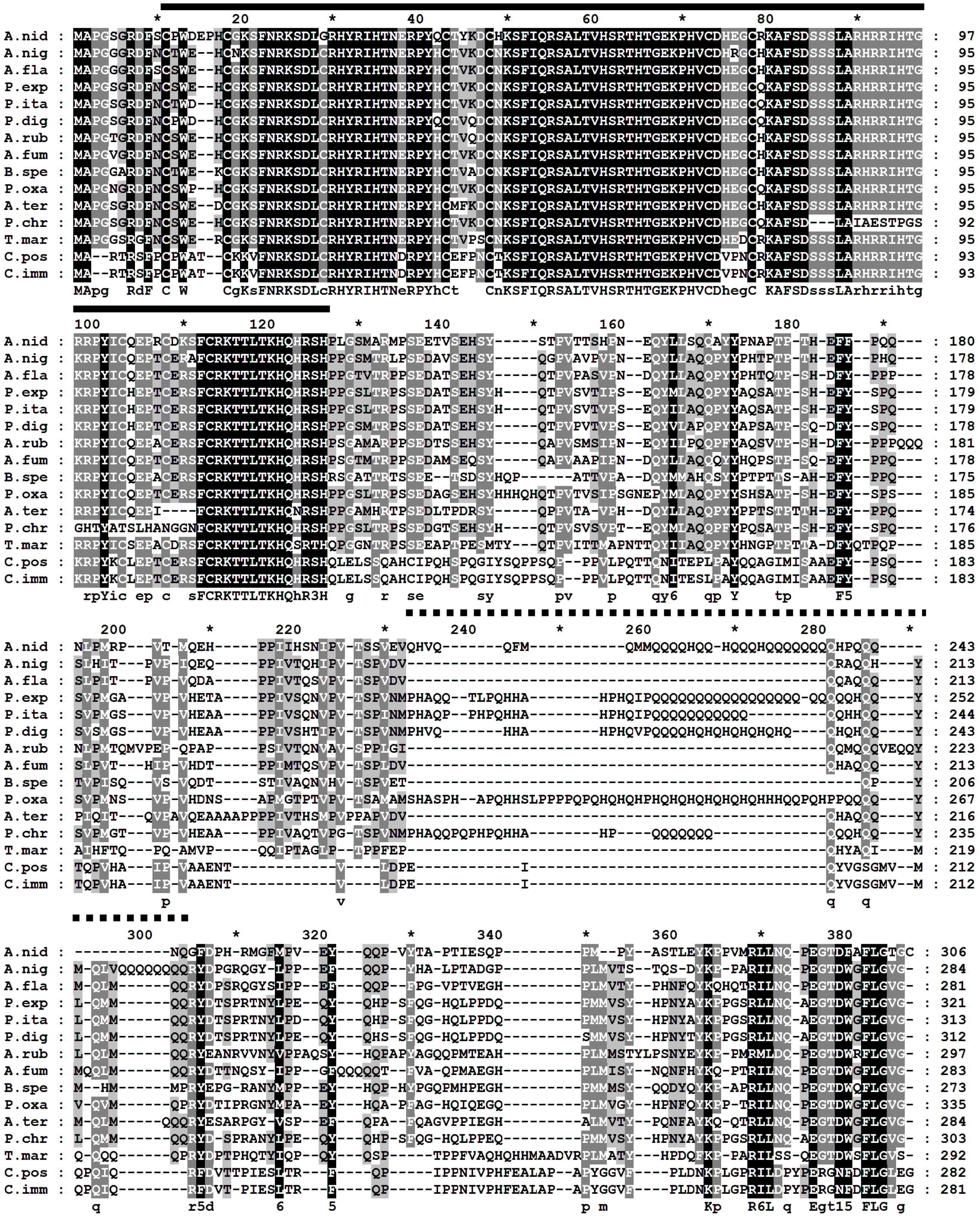

Supplementary Figure S6[36]

(B)

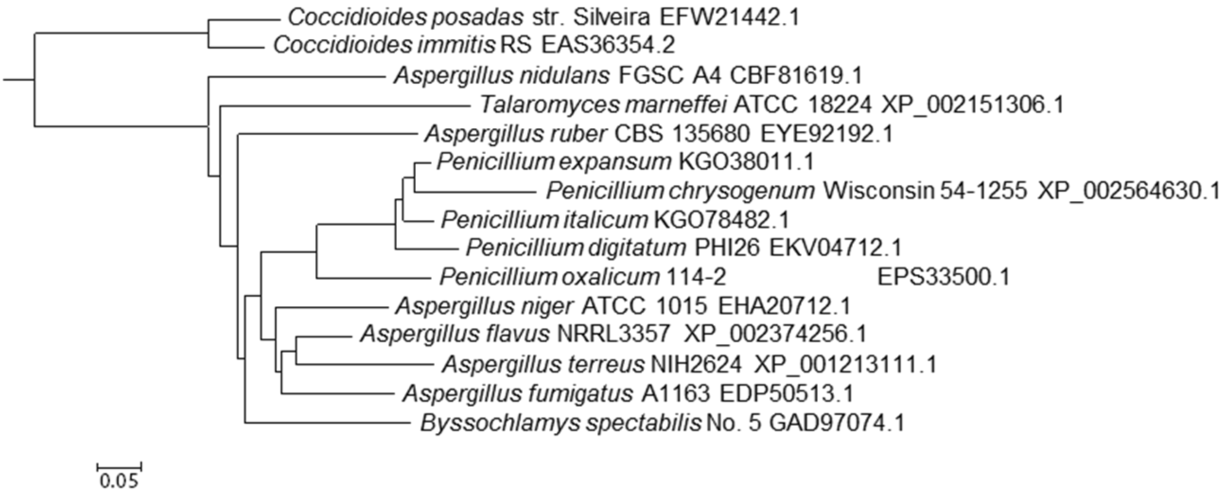

Supplementary Fig. S6. Analysis of the amino acid sequence of *A. nidulans* AslA. (A) Multiple alignment of the deduced amino acid sequences of AslA and its orthologues in different fungal species created Genedoc software (version 2.6.003; http://www.psc.edu/biomed/genedoc). The deduced amino acid sequence of AslA consists of 306 amino acids (Mr 35.6 kDa) that contain C_2H_2 zinc fingers (aa 11-126) near the N-terminus and a Gln-rich domain in the posterior portion (aa 209-245). Continuous dark line, C_2H_2 zinc finger domain; broken line, glutamine rich domain. Abbreviations: A.nid, *A. nidulans*; A.nig, *Aspergillus niger*; A.fla, *Aspergillus flavus*; A.fum, *Aspergillus fumigatus*; A.rub, *Aspergillus ruber*; A.ter, *Aspergillus terreus*; P.chr, *Penicillium chrysogenum*; P.exp, *Penicillium exspansum*; P.ita, *Penicillium italicum*; P.dig, *Penicillium digitatum*; P.oxa, *Penicillium oxalicum*; T.mar, *Talaromyces marneffei*; B.spe, *Byssochlamys spectabilis*; C.pos, *Coccidioides posadas*; C.imm, *Coccidioides immitis*. (B) Phylogenetic and molecular evolutionary analyses of AslA and its orthologues were conducted using MEGA software, version 3.1 (neighbor- joining method, with a bootstrap of 50,000 replicates and amino p-distance substitution model).
